# The spatial justice of school distribution in Jakarta

**DOI:** 10.1016/j.heliyon.2022.e11369

**Published:** 2022-11-03

**Authors:** Ahmad Aki Muhaimin, Ahmad Gamal, Michelle A.S. Setianto, Widya Laksmi Larasati

**Affiliations:** a SMART CITY CENTER, Universitas Indonesia, Depok 16425, Indonesia; b Master Program of Urban and Regional Planning, Universitas Indonesia, Depok 16425, Indonesia; c Department of Architecture, Faculty of Engineering, Universitas Indonesia, Depok 16425, Indonesia

**Keywords:** Spatial distribution, School, GIS, Accessibility, Equity

## Abstract

Education is a part of human basic needs, and one way to provide this need is through schools. Therefore, schools should be accessible to all members of society without exception. Jakarta, the capital of Indonesia, has 3899 primary and secondary schools to provide for over 1 million school-age children as of 2020. This research, however, will not dwell on the issue of capacity based on the number of demands and available schools, but rather the accessibility and fairness. Two methods: radius and road network, are used in this research with the addition of spatial analysis to measure the students’ walking accessibility level relating to the school zoning system regulations in Indonesia. The analysis result highlighted the variations in accuracy between the two methods, the coverage area between formal and informal settlements, and the spatial context of the accessibility. The result provides a comprehensive spatial analysis to support policymakers in future planning works.

## 1 Introduction

Education is a basic human right; thus, it should be available for everyone as stipulated in Article 26 of the Universal Declaration of Human Rights. Elementary-level education, in particular, is compulsory and should be free of all costs (United Nations, 1948). Education, generally alongside nutrition and health provisions, is always included as one of the humans' basic needs that are essential in eradicating poverty (Stewart, 1985; Potter and Lloyd-Evans, 2014). The argument made education facilities crucial, particularly for developing countries that struggle with sizable poverty rates (Cohen, 2006), hence highlighting the importance of the availability and accessibility aspects of the facility.

The availability aspect may have been responded to by building schools and providing supporting facilities such as books, equipment, and lecturers. However, the aspect of accessibility implies questioning whether those physical buildings, facilities, and human resources are distributed to those in the manner intended to ensure their well-being (Chatterton, 2010; Iveson, 2010; Williams, 2013). Yet research has argued that urban development and the socio-political situation have so far put this aspect of accessibility aside (Cohen, 2006; Potter and Lloyd-Evans, 2014).

From the development perspective, typically, urban development begins in the center and progresses to the outskirts. This pattern is followed by the development of public facilities such as hospitals, parks, and schools. Therefore, when an urban area has reached its peak development, many public facilities are centralized in the heart of the city (Cohen, 2006). Meanwhile, the socio-political aspect affecting public facilities' accessibility may come from the currently running government's policies, for example, the policy regarding cost. In developing countries, many primary education facilities are provided for free or subsidized to some point. Even though this alleviates the burden for parents, there are still costs for textbooks, study equipment or stationery, and uniforms, causing people from low-income households and those living in informal settlements to be unable to access the facility (Potter and Lloyd-Evans, 2014).

The situation is similar in Indonesia, a Southeast Asian developing country. The 1945 Indonesian Constitution stated that education should be accessible to all and the government then further established a nine-year compulsory basic education policy in 1989 (President of the Republic of Indonesia, 1989). The policy does not seem to be working very well. According to Statistics Indonesia, there are only 12.8% of heads of families from the low-income category between 2016 and 2021 finished their 9 years of compulsory school. The Indonesian president then re-established the regulation to cover 12 years of compulsory education (Indonesian Ministry of National Development Planning, 2014) or until the senior high school level. Yet the average percentage of heads of families from the low-income category who obtained their high school diploma after the policy establishment between 2016 and 2021 is still at 12.4%, half of those from families with better economic standing at 26.2% (Statistics Indonesia, 2022).

Aside from the compulsory education policy, the government seems to be working on the affordability aspect. For example, a stimulus program called Program Indonesia Pintar or Smart Indonesia Program (PIP) was launched by the President to assist children aged 6 to young adults aged 21 to finish their compulsory education. The program is prioritized for children from low-income families, orphans, natural disaster victims, and children with disabilities, according to the Ministry of Education and Culture Regulation No. 10/2020 (Indonesian Ministry of Education and Culture, 2020a). For general students, the government has also provided a school grant program known as Bantuan Operasional Sekolah or School Operational Assistance (BOS) to assist schools in the operational cost aspect (Indonesian Ministry of Education and Culture, 2021). The grant has made state schools practically free of charge while reducing private schools' students' fees.

It is safe to say that the government's efforts have covered the accessibility aspect from legal, regulatory, and financial perspectives. This prompted a question: what about the accessibility aspect from a spatial perspective? Related to this is the Indonesian school zoning system, where student acceptance to public schools is based on the proximity of the facility to the student's domicile. This zoning system is regulated at the local government level and described in detail. In Jakarta, for example, school zoning is regulated (and updated) through Governors' decree No. 440-year 2022. Here the information is as detailed as which neighbourhood in which sub-district is included in the school's service area and their prioritization level. Even though the description is very detailed, the basis is unclear. The ministry omitted the distance measurement from the zoning policy at the national level, considering the large variety of geographical or terrain conditions spread across the archipelago, and only emphasized the nearness of the education facility to the student's domicile as a requirement (Indonesian Ministry of Education and Culture, 2018a).

Distance to a public facility is a valid parameter to measure service coverage areas and increase the service rate. The Indonesian school zoning system, on the other hand, had the opposite effect: while it was initially around 70–90% in 2018, it dropped to 50–70% in 2019 (Indonesia Ministry of Education and Culture, 2018b, 2019). The decreasing schools' acceptance rate has become a burden for parents and their children in choosing their desired schools (Purwanti et al., 2019; Hoerudin, 2019), disturbing the children's accomplishments and limiting schools' innovation (Ismabela, 2019). Therefore, spatially, the schools' contribution should not only be measured by their coverage area but also by their accessibility (Muda, 2020).

The issue might be caused by the intention behind the school zoning system, which was mainly to ensure equitable service across the country. This system was meant to, amongst other things, reduce schools' exclusivity and discrimination, better distribute teaching materials and resources, and ensure the financial assistance programs are delivered to the targeted awardee (Indonesian Ministry of Education and Culture, 2018a). However, previous research has highlighted the potential benefit of a zoning system and students' easy access to school. Students living nearby (within the zone) will be able to travel to their school by foot instead of in motorized vehicles. This is crucial because underage students riding motorcycles is a common sight in Indonesia, which increases traffic jams as well as the risk of accidents because they are mentally underdeveloped. This is apparent in the police statistics, which stated that 73% of traffic accidents involved motorcycles, and 143 thousand offenders were under 17 years old (minimum age to receive a driving license) in 2021 (Pusiknas Bareskrim Polri, 2021). The statistic recorded only those involved in the traffic violation and accidents and is far smaller than the actual number of underaged students riding motorcycles to school, thus not really depicting the actual risk.

The reason why underage students drive motorcycles to school is that most parents permit them to do so for several reasons: security, convenience, and cost. The security issue is related to criminal acts that take place in public vehicles; convenience is related to route picking, and the cost is related to public transportation fees. Students riding motorcycles can cut travel costs and time commuting to schools while at the same time avoiding the crime risk of public vehicles. In addition, many motorcycle dealers offer low instalment pay with zero down payment (Umniyatun et al., 2021), which, despite the addition of gas, is still considered more efficient than riding public transportation, even for low-income families.

The government's attempt at a zoning system should be adequate to answer those worries. By studying in nearby schools, students will be able to travel by foot, thus reducing their exposure to public vehicle crime risk and traffic accident risk while lowering the cost and time spent travelling to and from school on a daily basis. Yet still, traffic accidents related to or caused by underaged students are still high, even after three years of the zoning system's establishment. This indicates two causes: first, the zoning was poorly calculated such that the travel distance is over the limit of comfort for travel by foot; second, the route is spatially inconvenient, thus unable to accommodate pedestrians.

The situation in Indonesia indicates that the provision of facilities for basic human rights, such as education, is not enough to be available. It demands good accessibility because the policy regarding availability would be useless if, at the end of the day, it was not able to provide equal service to those as intended (Hay, 1995). Equal access to educational facilities is one embodiment of social justice. The question is how to translate justice, which is initially an abstract concept, into a concrete space. Geographers have answered this by using “spatial justice” (Pirie, 1983) and referred to it as the consequential geography of the concept of justice (Soja, 2013). This perspective concretizes “justice” through its users' perspective of more than one parameter, such as distance, time, spatial quality, or the user's ability to use that access (Pirie, 1979, 1983). At least it should accommodate the minimum standards - both spatial and temporal (Hay, 1995). Therefore, this research attempts to utilize the spatial justice perspective in analyzing Indonesian schools' accessibility, in particular, as a critic of the zoning system. parameters, including the access route and spatial quality.

The research case study uses Jakarta because of the distinct wealth gap evident in the quality of formal and informal settlements. This is essential to highlight differences in planning quality. The city also has its share of slum areas, and a large portion of its demographic is school-aged children (Statistics Indonesia, 2020a). In accordance with previous research on the purpose of zoning systems, this research uses parameters adhered to by students travelling on foot. Additionally, this travel method is considered affordable to all students despite their economic background, thus enabling researchers to focus on spatial quality instead of the affordability of methods. This research will not compare transportation methods as has been done previously (e.g., Dai et al., 2019; Liao and Dai, 2022).

The study began with calculating service area using radius and road network methods based on walking distance, identifying the most facilitated formal and informal settlements to compare, and analyzing the spatial qualities of these settlements. The situation regarding the existing public service facilities is essential to future development planning. Therefore, it is necessary to consider both spatial aspects, such as location and access, and non-spatial aspects, such as user demographics, in the planning process (Yuan et al., 2017; Ashik et al., 2020). The assessments can determine urban facilities' adequacy level according to the spatial justice context (Martens et al., 2012; Lee and Miller, 2018). The result is a comprehensive view of spatial justice issues that can benefit planners, and related stakeholders, in ensuring that education is truly accessible to all without exception in the future.

## 2 Literature review

Research on access equitability has been done towards healthcare (e.g.: Rosero-Bixby, 2004; Cheng et al., 2015), schools and playgrounds (e.g.: Chin and Foong, 2006; Singleton, Longley, Allen, & O'Brien, 2011; Brindis et al., 2003; Shahraki et al., 2016; Dadashpoor and Alvandipour, 2020; Wen et al., 2018), city parks (Wu et al., 2017; Zhang et al., 2021) and public open or green spaces facilities (e.g.: Nicholls, 2001; Comber et al., 2008; Oh and Jeong, 2007; Csomós et al., 2021). The research used a similar method of temporal and spatial distance calculation relevant to the intended user signifying users as the main focus.

Equitable public facilities from users’ perspective were measured by availability within a specific area (Handy and Niemeier, 1997), proximity or distance (Ogryczak, 2000; Luo and Wang, 2003; Villarreal, 2013; Mahmoudi et al., 2019) or further added with spatial quality analysis (Brindis et al., 2003). The combination of methods brought the notion of spatial justice by acknowledging that the accessibility should be spatial too. In a sense that it is not enough that the facilities are there, but intended users should also be able to reach the facilities within expected time range (Ashik et al., 2020).

Spatial accessibility is often assessed by considering the amount of cumulative opportunity—i.e., the number of relevant facilities or destinations—reachable from a particular point within a certain distance or travel time (Morris et al., 1979; Vickerman, 1974). Spatial accessibility indicators are important because they show how effective and efficient a facility is by indicating how much time people must allot to reach a destination. The further a facility is from a target population, the less effective and efficient it will be (Savas, 1978; Social, 2020). The assessment of accessibility can calculate the anticipated demand for a facility in an area, which is also influenced by its maximum capacity. Gaps in accessibility level levels may indicate the inequitable distribution of facilities (Nicholls, 2001).

Non-spatial accessibility emphasizes non-geographic barriers such as social class, income, ethnicity, age, and disabilities (McLaughlin and Wyszewianski, 2002; Ashik et al., 2020) which are related to the concept of social justice. For example, in many cases, people with disabilities lack access to facilities that are not suitably constructed for use by this group. Likewise, people living below the poverty line may have minimal access to public service facilities located in the city center, especially if public transportation is unavailable. These non-spatial aspects are essential in measuring the spatial justice of public facilities, based on the region's socio-economic conditions (Polzin et al., 2014).

Many empirical studies have assessed the accessibility of various facilities for different demographic groups; some have looked simply for all facilities within a given radius (Yuan et al., 2017; Ashik et al., 2020; Dawod et al., 2013; Salarvandian et al., 2020), while others have used network analysis methods supported by geographic information systems (GIS) (Nicholls, 2001; Oh and Jeong, 2007; Salarvandian et al., 2020). The radius method can provide a simple estimation of travel distance because it assumes a straight-line transportation route from the point of departure to the facility (Nicholls, 2001). However, this is rarely the case for actual travel since one must use whatever roads are available and various obstacles, such as rivers or railroad tracks, may make the route more circuitous. Differences in the distance can lead to significant increases in time, cost, and effort for people with limited means and mobility and for the most vulnerable population groups, such as people with disabilities, the elderly, and young children (Ashik et al., 2020; Nicholls, 2001; Salarvandian et al., 2020). Often, the peripheral areas lack the number of and proper access to public service facilities, contrasting with the center of the city. These user groups are experiencing spatial injustice of access to public facilities; which means social justice has yet to be achieved.

Nicholls, Oh and Jeong, and Salarvandian adopted both the radius and network analysis methods to study the accessibility and equity of public service distribution (Nicholls, 2001; Oh and Jeong, 2007; Salarvandian et al., 2020). The simple radius method is used to ascertain the number of facilities and the proportion of the population in a selected area. Also, network analysis is performed to calculate travel distance along streets to different public services, based on the routes people use to travel from their residence to their destination (Ashik et al., 2020). The radius network is frequently applied for planning public service facilities to measure the coverage areas (Nicholls, 2001; Delamater et al., 2012). In the schools' zoning system in Jakarta (Pemerintah Provinsi DKI Jakarta, 2019), the score for distance parameters depends on linear or radius network to simplify how far the student's domicile to selected school locations (Dinas Pendidikan Provinsi DKI Jakarta, 2019). Although, as stated earlier, there is no exact basis of measurement.

## 3 Background of the study area

### 3.1 Informal settlements in Jakarta

This study used informal settlements data provided by City Without Slum or Kota Tanpa Kumuh abbreviated as KOTAKU and Statistics Indonesia (City Without Slum, 2017; Statistics Indonesia, 2017). Slums often called as a dysphemism for “informal settlement” (Dovey et al., 2020). As a definition, the term ‘slum’ refers to inner-city residential areas having progressively overcrowded, dilapidated, unsafe and inadequate access to basic infrastructure and public services (UN-Habitat, 2003). While the term ‘informal settlement’ refers to illegal residential places built by lowest-income groups called squatters without formal permission of the land ownership and improper building categorized as slums referred to as squatter settlements (UN-Habitat, 2003). Statistics Indonesia and KOTAKU also define the rate of slums according to (1) poorly built and unregulated residential buildings, (2) poor road network safety and standards, (3) poor drainage quality, (4) poor drinking water services, (5) lack of a sewage system, (6) poor home waste management, and (7) lack of fire protection for most buildings and infrastructure. The assessment of the area was based on field observation, surveys, and government agencies' data (City Without Slum, 2017; Statistics Indonesia, 2017). Thus, this study used informal settlements as slum areas and formal settlements as non-slum areas in Jakarta. The slum area mapping in KOTAKU is based on criteria provided by the Ministry of Home Affairs and conducted by the Ministry of Public Works and Public Housing (Indonesian Ministry of Public Works and Public Housing, 2010). The smallest administrative area is a village, which consists of several neighborhoods known as Rukun Warga (RW). A slum can consist of one or more RWs. At the same time it is possible for a village to have both slums and non-slums neighborhoods. In total, there are 164 villages in Jakarta consisting of 36 in the West, 33 in the Central, 26 in the North, 32 in the East, and 37 in the South (Statistics Indonesia, 2017).

**Figure 1:**
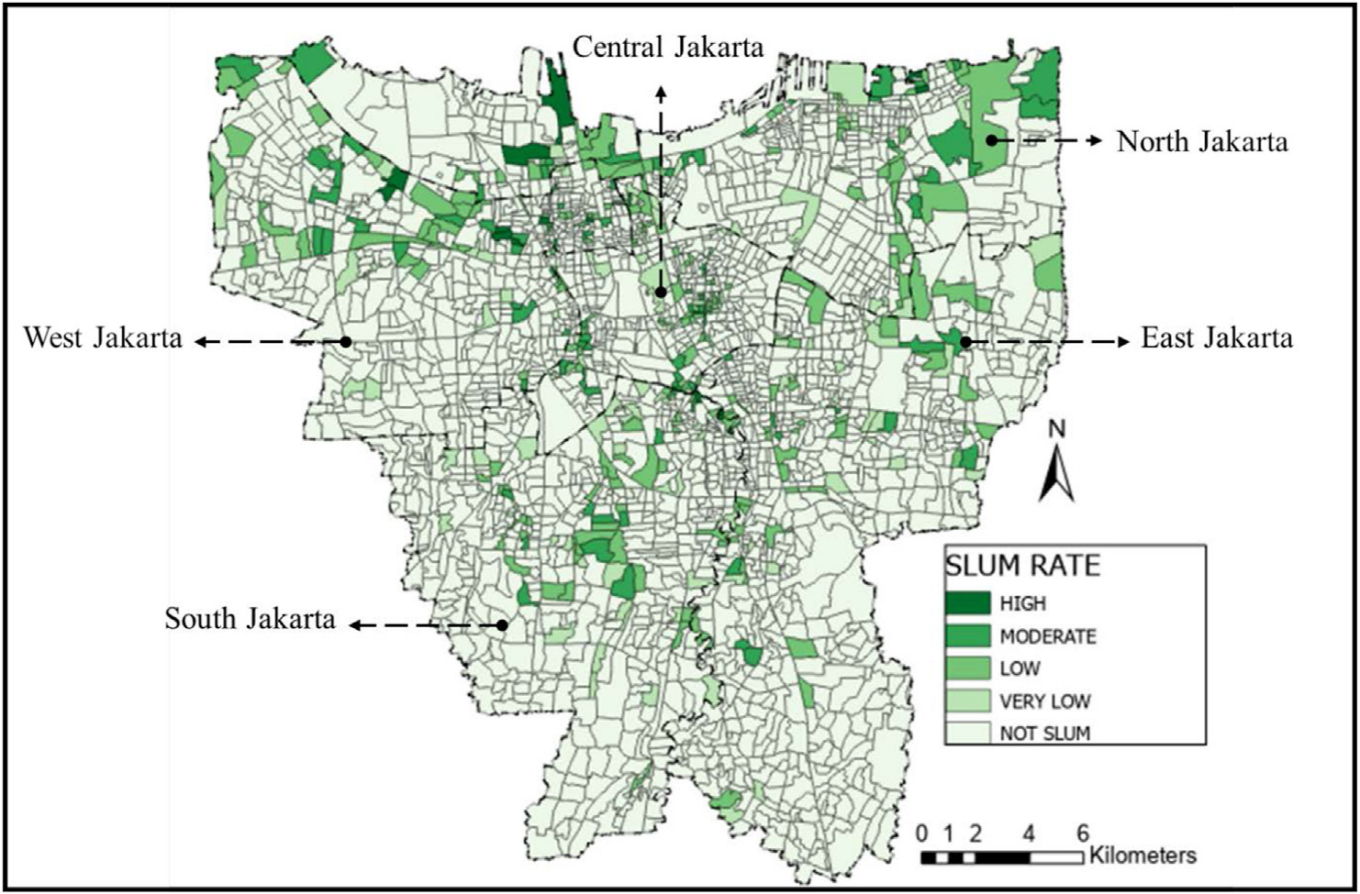
Distribution of slum area in Jakarta. Source: KOTAKU; modified by the authors.

Figure 1 is a KOTAKU's settlement map with slight modifications to illustrate the location of the formal and informal settlements. The formal settlements are colored in light green, while the informal settlements are colored in darker colors. The range of color is respective to the slum rate, of which the darker the color is, the higher the slum rate. Moreover, Table 1 is a calculated area of formal and informal settlements using ArcGIS 8.0 where the area of informal settlements is a cumulative of very-low slum areas to high slum areas and formal settlements area only represent the non-slums area in each city.

**Table 1:** List of the area of the formal and informal settlements.

Regional Area	Total Area (km^2^)	Formal settlements (km^2^)	Informal settlements (km^2^)
West Jakarta	125,43	102.52	22.91
North Jakarta	139,90	105.87	34.03
Central Jakarta	47,94	39.80	8.15
East Jakarta	185,13	163.54	21.58
South Jakarta	144,85	125.60	19.25
Total	643.25	537.33	105.92

A slum area is also identified from its population density of more than 200 people per square kilometer (Indonesian Ministry of Public Works and Public Housing, 2006). The types of residences may consist of a house occupied by a single large family or a small building occupied by several families living together. The houses may be equipped with a shared kitchen, toilet or water access, and narrow corridors and passages between rooms. These settlements' high building density of over 100 buildings per hectare (Indonesian Ministry of Public Works and Public Housing, 2006) also reduces infiltration areas or green open spaces. In turn, the minimal infiltration area hampers water flow, making the areas prone to flooding, especially in locations adjacent to the rivers and sea.

### 3.2 Schools in Jakarta

The number of schools and students including private and public schools based on the educational levels from elementary to high school in Jakarta are listed in Table 2. In total, there are 2,350 elementary, 1,061 junior, and 488 senior high schools spread across Jakarta. It is clearly seen that East Jakarta has the most schools in total and at each educational level except in junior high schools, while Central Jakarta has the least schools in total and at each educational level (Statistics Indonesia, 2020b, Statistics Indonesia, 2020c, Statistics Indonesia, 2020d). Based on the number of students in each educational level, it can confirm that the number of schools adjusts to number of students in which the higher the number of students, the more schools are available in one city. However, it is not known whether the school's spatial distribution and accessibility are adequate or not.

**Table 2:** List of the number of schools and students in Jakarta.

Regional Area	Elementary		Junior High		Senior High	
	Unit	Student	Unit	Student	Unit	Student
West Jakarta	599	185.166	278	79.761	116	32.118
North Jakarta	345	132.539	193	58.888	89	25.581
Central Jakarta	279	80.522	112	42.145	56	19.710
East Jakarta	639	247.836	261	108.708	123	55.124
South Jakarta	488	166.193	217	78.855	104	41.594
Total	2350	812.256	1061	368.357	488	174.127

## 4 Methodology

This research primarily utilized GIS information available from a government, NGO, and open-source websites as the object of analysis. Field observation was not possible because the research was conducted during the pandemic. Therefore, the information regarding physical condition of accesses was retrieved from Google applications such as Map and Street View. A detailed list of information and sources is available in the following Table 3.

**Table 3:** Data types and sources.

Data Type	Year	Source
Road network	2020	OpenStreetMap (OSP) Indonesia
Boundaries (areas, lots, rivers, etc.)	2020	BPDB Jakarta and OpenStreetMap (OSP) Indonesia
Education facility points (elementary, junior high, and senior high schools)	2020	OpenStreetMap (OSP) Indonesia
Access points (entry and exit)	2020	Observation through Google Map and Google Street View
Informal settlements' locations	2017	KOTAKU and Statistics Indonesia

The GIS data were then analysed, together with distance measurements, to provide information on the educational facilities' service area coverage. Accessibility is defined as the distance that individuals had to travel to reach a particular school from their residence (Tsou et al., 2005). Therefore, the study collected various recommendations and standards on this issue, including walking distance, distance radius, and the on-foot travel duration separated by the educational facilities’ level. The recommendations on the comfortable walking distance for the general public were added as a comparison. The details are available in Table 4. The analysis does not cover preschool/kindergarten and university or its equals as they are not included in the twelve years compulsory education programs in Indonesia by Local Regulation DKI Jakarta Province No. 8 years 2006 about Education System (Pemerintah Provinsi DKI Jakarta, 2006).

**Table 4:** Travel distance recommendations.

Actors	School level	Distance (m)	On-foot travel duration (min)	References
Students	Preschool/kindergarten	400	5	Barton et al. (2003), Perry et al. (1929)
	Elementary/primary	500	5–10	Azmi et al. (2012), Olson (2010). Stein (1942)
		500–600	5	Perry et al. (1929)
	Junior high	500	5–10	Azmi et al. (2012), Olson (2010), Stein (1942)
	High school	1000	15–20	Barton et al. (2003)
General public	n/a	1000–2000	10–15	Azmi et al. (2012), Barton et al. (2003)

After defining the educational facilities' area coverage, the study continues with analysing accessibility and social equality. From the literature review, the research compiled two dimensions and their respective attributes to measure accessibility. A scoring index was created to ease the calculation, as presented in Table 5. Meanwhile, the social equality analysis is done based on KOTAKU map that provides information on settlement types. The settlement types were classified according to the households’ income levels. This research opted for the most well-facilitated districts informal and informal settlements to better illustrate the social equality level.

**Table 5:** Accessibility indicators and scoring index.

Dimension	Attribute	Scoring Value
Safety	Presence of sidewalk	1 = Satisfactory 0 = Acceptable −1 = Unsatisfactory
	Continuity of sidewalk	
	Sidewalk width (1.5–2 m)	
	Separation from traffic: *the presence of barrier or level differences between sidewalks and the road*	
Comfort	Levelness	
	Obstruction	
	Protection from weather	

## 5 Results

### 5.1 The educational facilities’ service area coverage ratio

This section provides the schools' service areas and the area coverage comparison between formal and informal settlements in the five districts of Jakarta. The measurement of distance is adjusted according to the level of the educational facility, as presented in Table 3. The coverage ratio (CR) per district is calculated by distributing the school's service area coverage (As) per the district's total area (Ad), as illustrated in Eq. (1).

(1) $$CR\;\left(\%\right):\;\frac{As}{Ad}\times 100$$

The service area coverage was also used to calculate the ratio of facilitated areas at formal (CRf) and informal settlements (CRi) by tracing them onto the settlement map provided by KOTAKU. The coverage ratio of schools at formal and informal settlements is similarly calculated per district as illustrated in Eq. (2), the formal settlement coverage ratio (CRf) is calculated by distributing the school's service area coverage for formal settlement (Asf) per district's total area of formal settlements (Adf), while the informal settlement coverage ratio (CRi) is calculated by distributing school's service area coverage for formal settlement (Asi) per district's total area of formal settlements (Adi).

(2) $$CR\;\left(f,i\right)\;\left(\%\right):\;\frac{As\;\left(f,i\right)}{Ad\;\left(f,i\right)}\times 100$$

**Figure 2:**
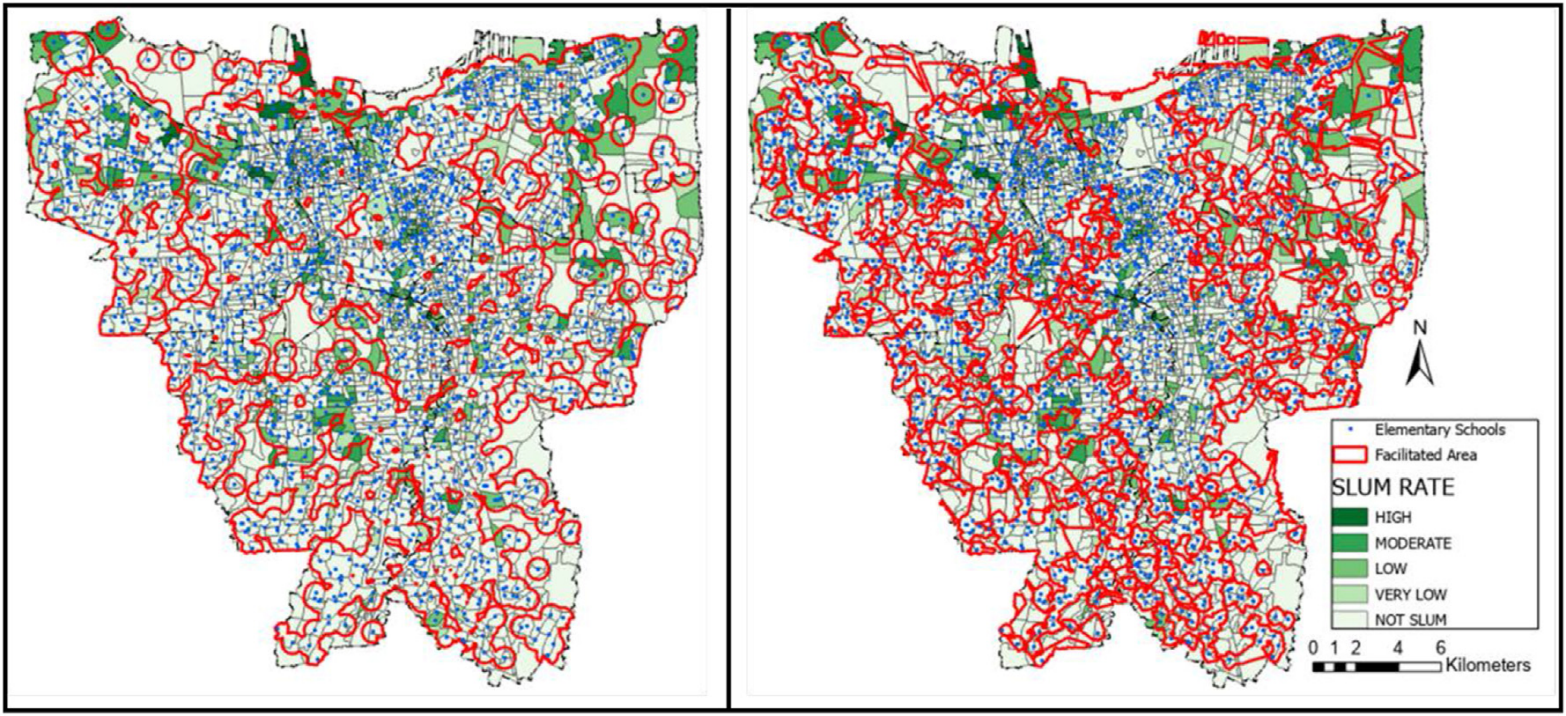
Elementary school distribution mapping in Jakarta based on (a) radius method and (b) road network method. Source: ArcGIS; modified by the authors.

**Figure 3:**
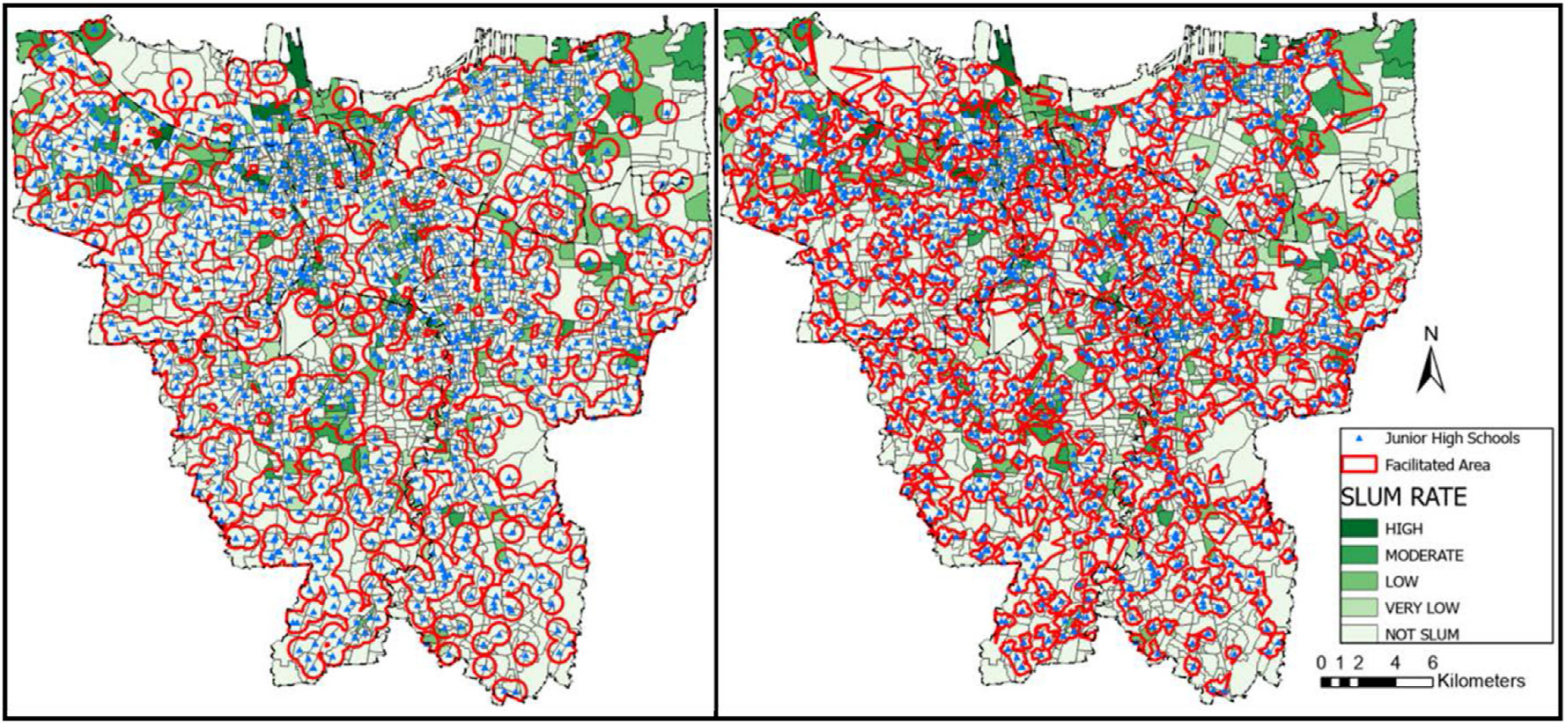
Junior high school distribution mapping in Jakarta based on (a) radius method and (b) road network method. Source: ArcGIS; modified by the authors.

**Figure 4:**
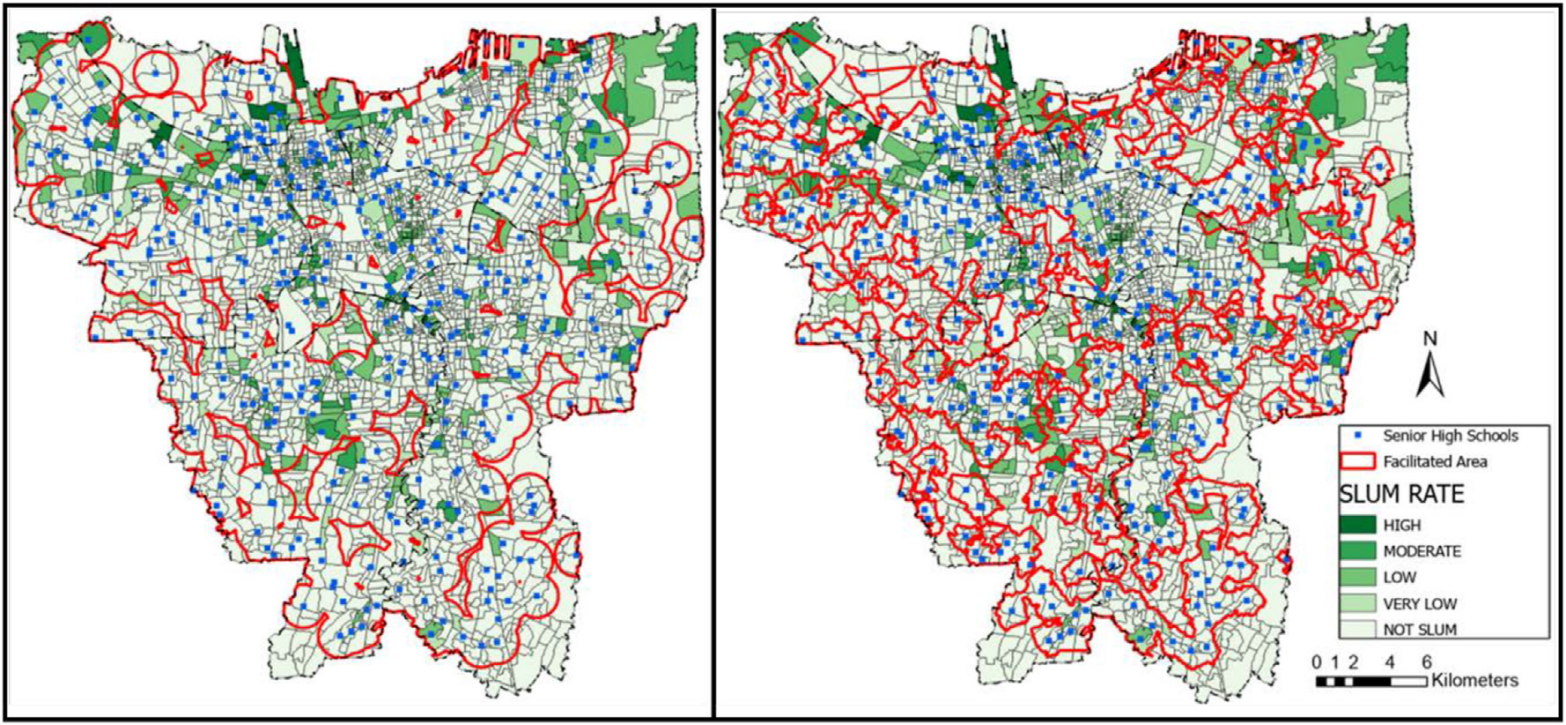
Senior high school distribution mapping in Jakarta based on (a) radius method and (b) road network method. Source: ArcGIS; modified by the authors.

The service area coverage data was calculated using the radius and road network method. The radius method relies on the recommended traveling distance measured outward from the location of the facility with no regard to access. Meanwhile, the road network method applied the recommended traveling distance from the facility's location outward based on available accesses. Figure 2, Figure 3, and Figure 4 present the result of service area coverage analysis per educational level in both methods. It is understood from the image, coloured red, that the radius method has produced a wider area coverage compared to the road network.

Table 6a and b display the service area coverage and ratio per district in figures. In this table, Central Jakarta is the most well-facilitated district in general according to radius and road network analysis, while North Jakarta is the least. Although North Jakarta has the least coverage ratio on average, the most contrasting difference in radius and road network analyses is apparent in the Junior High schools in East Jakarta. In this district, the radius method provides a 60% coverage while the road network shows less than 40%. This number indicates that accessibility is essential to make a district well-facilitated.

**Table 6:** Service area coverage and ratio per educational facilities’ level.

a. Service area coverage (km^2^)						
	Elementary		Junior high		Senior high	
Regional Area	Radius (km^2^)	Road network (km^2^)	Radius (km^2^)	Road network (km^2^)	Radius (km^2^)	Road network (km^2^)
Central	43.31	37.18	37.89	25,62	47.39	38.93
North	85.17	81.23	67.92	46,13	99.05	67.45
South	109.61	88.69	95.43	61,59	65.88	76.17
West	108.97	87.78	93.59	60,07	114.74	47.89
East	126.08	101.33	112.57	72,57	60.81	105.82
**Total**	**473.13**	**396.21**	**407.39**	**265.97**	**528.05**	**373.35**

b. Coverage ratio (%)						
	Elementary		Junior high		Senior high	
Regional Area	Radius (%)	Road network (%)	Radius (%)	Road network (%)	Radius (%)	Road network (%)
Central	90.33	77.55	79.03	53.43	98.84	81.21
North	60.88	58.06	45.55	32.97	70.80	48.21
South	75.67	61.23	65.88	42.52	81.81	52.59
West	86.88	69.98	74.61	47.89	91.47	67.74
East	68.10	54.74	60.81	39.20	80.15	57.16
**Total**	**73.53**	**61.59**	**63.33**	**41.35**	**82.09**	**58.04**

The radius network analysis showed the educational facilities coverage ratio in Jakarta, except Kepulauan Seribu district, as low. However, Table 6b also reveals the discrepancy. For example, there is an over 30% difference in Senior High school coverage ratio between Central and North Jakarta: in which the former is facilitated for more than 80% while the latter is less than 50%. Interestingly, according to Table 6a, the services are of schools in Central Jakarta is the lowest amongst the five districts. This percentage indicates that the service area calculation result is not necessarily parallel with the coverage ratio. There are several factors, including the number of schools, the location of the schools, the district's area, the availability of access, and the land use. The availability of access, amounts of schools, and strategic location dispersion can increase the coverage ratio.

The coverage analysis result was placed above the KOTAKU's settlement map to investigate the coverage ratio per formal and informal settlements. The result is presented in Table 7a and b similar to the coverage ratio per district in Table 6b prior, the radius method generally provides a more favourable result than the road network. Despite the large discrepancy, the formal and informal settlements at Central Jakarta district are the most well-facilitated. Interestingly, both radius and road network method provide a higher coverage ratio for the informal settlements than formal settlements in this district. This result is similar with the statement from Cohen that the public facility and infrastructure begins at the center before spreading to the peripheries in a city or province (Cohen, 2006). Another reason is due to the Central Jakarta is the smallest cities comparing other cities. It means that either road network method or radius method obtain higher coverage ratio in both settlements.

**Table 7:** Formal and informal settlements coverage ratios.

a. Comparison of formal and informal settlements coverage ratio (radius method)						
	Elementary coverage ratio (%)		Junior high coverage ratio (%)		Senior high coverage ratio (%)	
Settlement/Regional Area	Formal	Informal	Formal	Informal	Formal	Informal
Central	88.54	99.07	76.74	90.23	98.61	100.00
North	62.81	54.89	51.40	39.66	75.68	55.62
South	75.53	76.59	66.08	64.61	81.74	82.51
West	88.17	81.08	76.66	65.47	92.55	86.64
East	67.99	68.95	61.59	54.89	80.36	78.60
**Total**	**74.10**	**70.76**	**64.63**	**56.77**	**83.44**	**75.26**

b. Comparison of formal and informal settlements coverage ratio (road network method)						
	Elementary coverage ratio (%)		Junior high coverage ratio (%)		Senior high coverage ratio (%)	
Settlement/Regional Area	Formal	Informal	Formal	Informal	Formal	Informal
Central	75.34	88.32	50.89	65.86	78.40	94.91
North	59.56	53.42	34.67	27.68	53.42	32.00
South	61.62	58.68	42.02	45.78	53.75	44.97
West	71.85	61.60	49.53	40.57	68.48	64.47
East	54.85	53.84	39.75	35.06	56.53	61.97
**Total**	**62.12**	**58.92**	**41.97**	**38.20**	**59.17**	**52.32**

This pattern is repeated in the South Jakarta district, only with a slight variation. The elementary and senior high coverage ratio based on radius method and junior high based on road network method are higher at informal settlements in the district. Meanwhile, the contrasting results are presented in other categories.

The coverage ratio analyses have shown the broadness of schools’ service areas in their respective district. Despite the road network method providing a more realistic result, as in really put access into consideration instead of merely drawing a radial image of distance, further analysis on the spatial aspect is still required to assess the accessibility. The spatial analysis concerns seven attributes of two dimensions: safety and comfort listed in Table 4. Since the coverage focuses on walking distance appropriate to each educational level, the spatial analysis will concentrate on the pedestrians.

Previously mentioned that the research would commence a spatial analysis upon well-facilitated formal and informal areas. The coverage ratio analyses pointed to Central Jakarta as the best-facilitated district with its three formal settlements: Gunung Sahari, Pasar Baru, and Cempaka Putih Timur, and three informal settlements: Karet Tengsin, Bendungan Hilir, and Menteng. The result is available in the following section.

### 5.2 Accessibility evaluation of formal settlements spatial analysis in formal settlements

**Figure 5:**
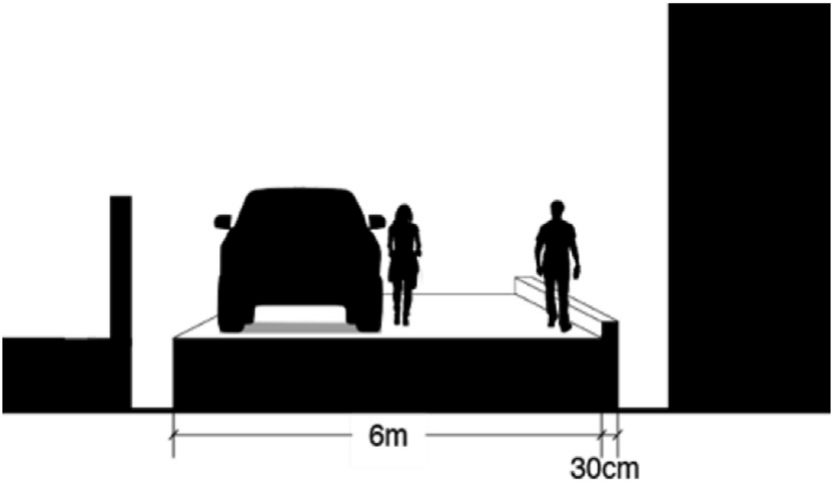
Access at Kelurahan Gunung Sahari Selatan, Central Jakarta. Source: Google Street View, modified by the authors.

**Figure 6:**
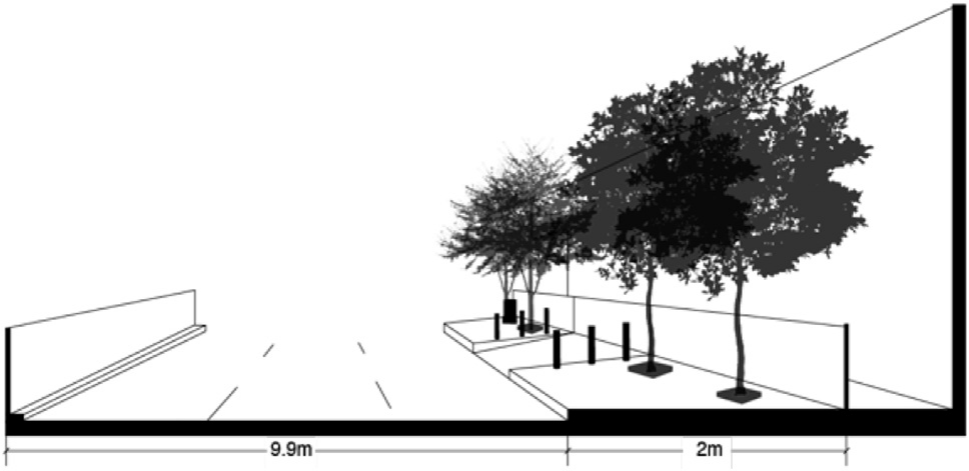
Access at SMPN 5 Jakarta, Central Jakarta. Source: Google Street View, modified by the authors.

The spatial analysis began with measuring the section drawing of accesses to schools in the area, which are made based on authentic images from Google Street View. Figure 5 illustrates access to elementary schools located in a residential area of Gunung Sahari Selatan administrative village. There are two types of neighborhoods locations: those in a residential complex and those adjacent to the major road. The elementary schools are both located inside a housing complex. Figure 6 shows that the dedicated pedestrian access to the schools in this village is non-existent despite being a formal settlement and occupied by upper-middle-class citizens. There is only 6-meter road access for both pedestrians and two-way traffic. Parking vehicles on the roadsides in front of the school often further reduced the available space. The absence of sidewalks might be because the schools are located in a housing complex, and therefore traffic is expected to be low in density and speed. Likewise, the neighborhoods adjacent to major roads can enjoy wide sidewalks because of their location. Nonetheless, this sidewalk access is not continuous to the schools and therefore is considered insufficient.

Junior and senior high schools in Central Jakarta are primarily located in the north and eastern parts of the district alongside most of the residential areas. The rest of the district mainly consist of commercial, business, and government buildings. Pasar Baru in the north is the example of a well-facilitated formal settlement of this district. The pedestrian access to a high school in Pasar Baru village is shown in Figure 6. There is a two-meter-wide sidewalks elevated by 30 cm from the road surface with dividers to prevent motorcycles from entering. The sidewalks are free from parked vehicles, making them accessible and safe for pedestrians. Trees are planted in concrete boxes, 40 × 40 square centimeters in size, thus able to provide comfortable shade for pedestrians without taking up too much space. However, it should be noted that Pasar Baru is also mainly a commercial area, which explains the existence of three lanes road in the image.

**Figure 7:**
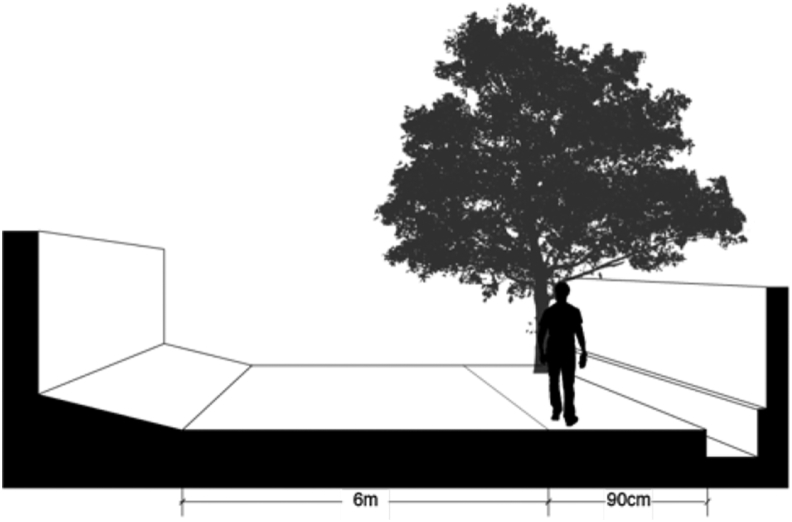
Access at Cempaka Putih Timur Street, Central Jakarta. Source: Google Street View, modified by the authors.

Another well-facilitated formal settlement in Central Jakarta is Cempaka Putih Timur. Figure 7 shows a typical road section in a residential area in this village, which, as Gunung Sahari Selatan, is also not equipped with sidewalks. However, there are 90-cm-wide culvert-covered paths adjacent to the road that pedestrians can use, although they also have to use the road when encountering obstructions such as electrical panels and potted plants, thus increasing their safety risk rate.

Table 8 presented the index calculation result of the three well-facilitated administrative villages in Central Jakarta District. Pasar Baru scored the highest in both safety and comfort dimensions, while Gunung Sahari received minus points. Meanwhile, Cempaka Putih Timur is generally considered as acceptable, as some attributes such as sidewalk's barrier and levelness negate the satisfactory sidewalk and weather protection points.

**Table 8:** Index scoring of accessibility for formal settlements in Central Jakarta.

Attribute/administrative village	Gunung Sahari	Pasar Baru	Cempaka Putih Timur
**Sidewalk safety**			
Presence	-1	1	1
Continuity	-1	1	0
Width	-1	1	0
Barrier/elevation	-1	1	-1
**Sidewalk comfort**			
Levelness	-1	1	-1
Obstruction	0	1	0
Weather protection	-1	1	1
**Total score**	**-6**	**7**	**0**

### 5.3 Accessibility evaluation of informal settlements

**Figure 8:**
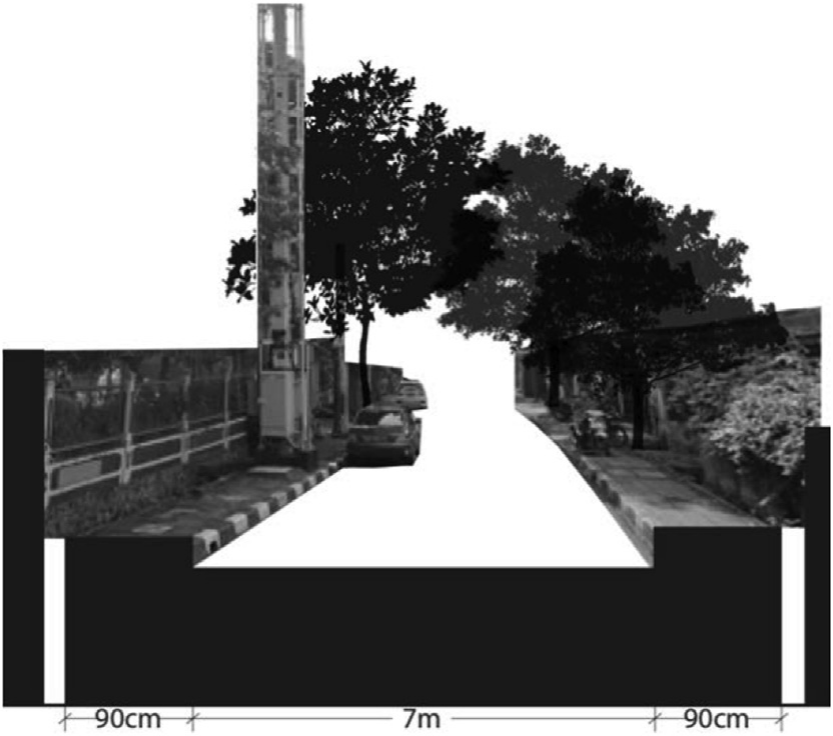
Access at Bendungan Hilir Village. Source: Google Street View, modified by the authors.

Following the spatial analysis of accesses in well-facilitated formal settlements is the analysis of informal settlements located in the Central Jakarta district. The informal settlements are part of Karet Tengsin, Bendungan Hilir, and Menteng administrative villages. The three informal settlements are located at the edge of the Ciliwung River. The accesses in these areas face similar problems of either the sidewalks are absent or discontinued. Sidewalks are available only on the major road, yet they are only one meter wide or less and occupied with various obstacles. Table 9 shows the index calculation result of the selected informal settlements while, Figure 8 illustrates the access in Bendungan Hilir. Although there are sidewalks on both sides of the road, each is only 90-centimeter wide and is immediately obstructed by trees, other structures, or parking vehicles.

**Table 9:** Index scoring of accessibility for informal settlements in Central Jakarta.

Attribute/administrative village	Bendungan Hilir	Menteng	Karet Tengsin
**Sidewalk safety**			
Presence	1	-1	-1
Continuity	0	-1	-1
Width	0	-1	-1
Barrier/elevation	1	-1	-1
**Sidewalk comfort**			
Levelness	1	-1	-1
Obstruction	-1	-1	-1
Weather protection	1	0	-1
**Total score**	3	-6	-7

**Figure 9:**
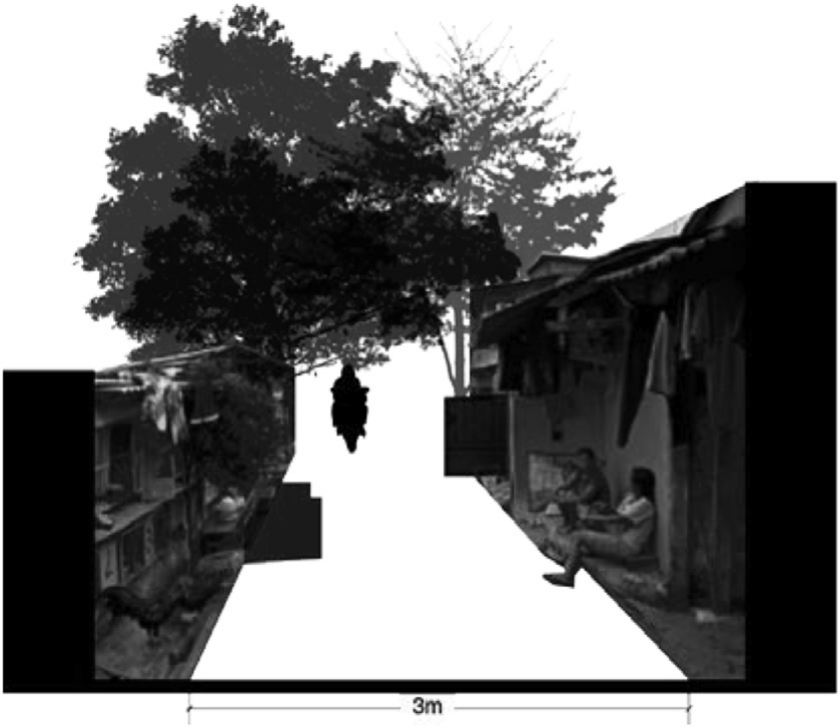
Access at Menteng Village, on the banks of the Ciliwung River. Source: Google Street View, modified by the authors.

**Figure 10:**
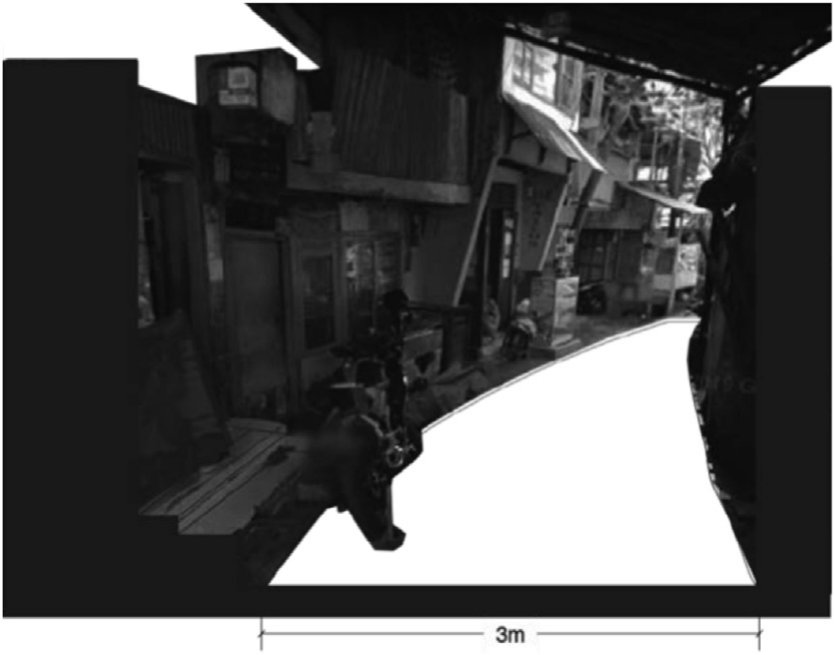
Access at Karet Tengsin. Source: Google Street View, modified by the authors.

Figure 9 illustrates the access at Menteng village. There is only one three-meter wide road that accommodates pedestrians, traffic, parking vehicles, trees, and random properties of the inhabitants. Figure 10 of access on Karet Tengsin village illustrates a similar condition. Both roads are narrow and risky. The houses in both villages generally have no front yard; therefore, the already narrow road is treated as one. The roads are considered as the garage, socializing place, and commercial store-front. Homeowners park their vehicles, conduct socializing events, store their belongings, or open their businesses on the roadside.

From the index scoring, the informal settlement in Bendungan Hilir scored the highest at three points. This informal settlement is facilitated with sidewalks as it is located at a major road, albeit the many obstructions. Meanwhile, the informal settlements in Menteng and Karet Tengsin villages scored minus six and minus seven, respectively. The spatial analyses produced a similar result between formal and informal settlements. Despite the different road measurements, both show that the settlements located at major roads score better because the sidewalks are present at the very minimum.

## 6 Discussion

**Figure 11:**
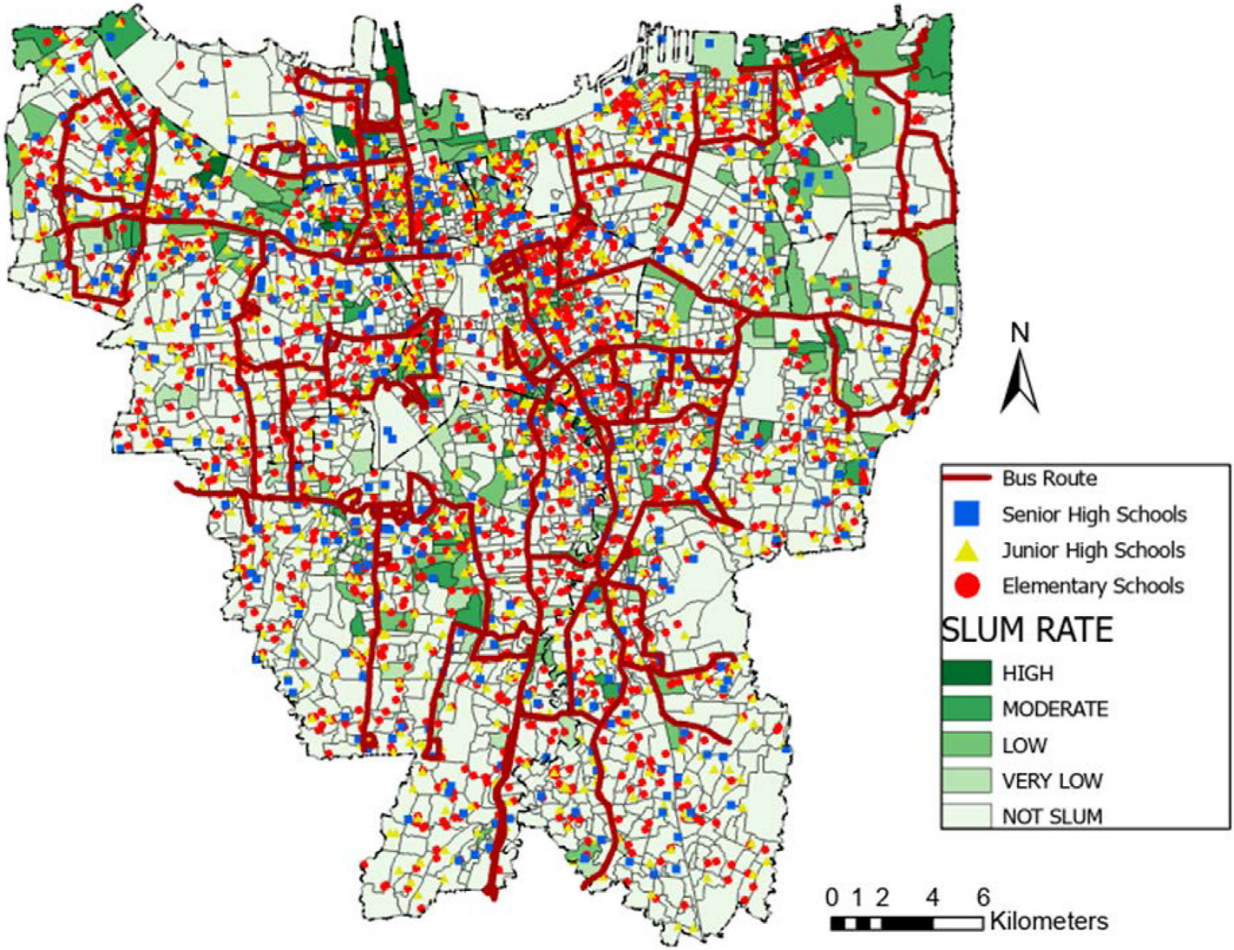
School bus route in Jakarta. Source: Moovitapp, modified by the authors.

The implication of schools' acceptance zoning system evoked protests from students' parents, particularly in Jakarta (Sirojudin et al., 2020). The school's distribution map in Figures 2, 3, and 4 showed the inequality, especially since more facilities are available in the formal settlements. Only 50–75% of the informal settlements are near enough to be included in a school's zone. Furthermore, students whose domicile location is covered by multiple zones have more options and a bigger chance of being admitted to the desired school than those who are remote. The unequal dispersion of the facilities already increases the gap between lower-income citizens living in informal settlements with their counterparts. This result is the opposite of the purpose of the zoning system: to remove the ranking gap between schools (Purwanti et al., 2019; Hoerudin, 2019; Ismabela, 2019; Sirojudin et al., 2020). In terms of equitable access to all levels of society, this zoning system greatly facilitates all communities, both informal and formal settlements, to have the same access and freedom to school by walking. However, from the results, it was found that students in informal settlements did not get good access to schools. Residents in this area are mostly in middle to lower economic levels, so the students prefer to walk or use the free school bus. Moreover, not all school bus is going through the informal settlements, as shown in Figure 11.

There are several ways to improve students’ access on foot to school, especially in informal settlements. The first way is by improving the quality of planning and road construction by developing the design regulations for road construction around schools, considering the aspect of safety and convenient access to school. With this, parents will not worry about the safety of their children when walking to school and will not be burdened with the cost of transporting their children to school. In addition, this study has a limitation that only evaluates the distance based on walking accessibility, so further research related to spatial justice based on commuting distance also needs to be analysed in the future. This will increase recommendations regarding how to build roads around schools where students have long commuting distances. In addition, with this research, the route of the free school bus facility can be improved so that it can reach all students, whether from formal settlement or informal settlement.

In addition, this research was carried out during the Covid-19 pandemic, so there was a policy change from the Ministry of Education regarding learning methods from school to students' homes or online learning (Indonesian Ministry of Education and Culture, 2020b). Although this does not have an impact on the school zoning system. But, with the school zoning system, students affected by Covid-19 in schools close to their homes can easily be tracing the students’ mobility (Komisi Perlindungan Anak Indonesia, 2020).

## 7 Conclusion

The radius and road network analysis of education facilities' accessibility in Jakarta produced contrasting results. Generally, the radius method provides a more favorable 63–82% coverage ratio for all education levels, while the road network method had 41–62%. There is approximately a twenty percent rate difference in the results. The highest coverage area is located in Central Jakarta, albeit the least number of schools. The district is equipped with well-connected roads, therefore, further increasing its accessibility. However, the comparison result of spatial analysis of well-facilitated formal and informal settlements in Central Jakarta shows that formal settlements still scored better than the informal despite being located in the same district.

These findings indicate that this city's development is more well-thought in the center than in the peripheries. Unfortunately, even with such well-thought planning, the spatial treatment disparity between formal and informal settlements is still very much noticeable. The significant result gap between the two methods also suggests that the radius method is unsuitable for planning as it does not comprise the actual on-field data. Furthermore, the road network method should also involve spatial analysis when assessing accessibility. The overall result indicates that the education facility in Jakarta city is not well distributed and far from being accessible in terms of distance and spatial quality.

The strength of this study is that this study shows a simple spatial analysis but produces a complex and crucial analysis related to spatial justice in the distribution of schools in Jakarta. Thus, this study can assist planners in achieving the ideal spatial justice of education facilities in the future. Planners and related stakeholders can utilize directly simple spatial methods such as the road network analysis to determine future development targets and overcome the social gap between society, particularly for educational facilities. However, the authors understand that in this study, some shortcomings or limitations can be developed in the future. This study limits the input parameter, focusing on the walking accessibility of students. Future research can improve the spatial analysis by expanding the analysis such as the travel accessibility with various of vehicles, method such as kernel density techniques, autoregressive models, census geographies, or travel time analysis, and creating comprehensive city data to assist urban development planning further. Finally, the city needs to achieve spatial justice to support the government in providing education facilities accessible to all as stipulated in the constitution.

## Declarations

### Author contribution statement

Ahmad Aki Muhaimin: Conceived and designed the experiments; Performed the experiments; Analyzed and interpreted the data; Contributed reagents, materials, analysis tools or data; Wrote the paper.

Ahmad Gamal: Conceived and designed the experiments; Contributed reagents, materials, analysis tools or data; Wrote the paper.

Michelle A. S. Setianto: Conceived and designed the experiments; Performed the experiments; Analyzed and interpreted the data; Wrote the paper.

Widya Laksmi Larasati: Analyzed and interpreted the data; Contributed reagents, materials, analysis tools or data; Wrote the paper.

### Funding statement

Mr. Ahmad Gamal was supported by 10.13039/501100014823Direktorat Riset dan Pengabdian Masyarakat Universitas, Indonesia [Grant # NKB-1750/UN2. RST/HKP.05.00/2020].

### Data availability statement

Data will be made available on request.

### Declaration of interest’s statement

The authors declare no conflict of interest.

### Additional information

No additional information is available for this paper.
